# Evaluation of whole and core genome multilocus sequence typing allele schemes for *Salmonella enterica* outbreak detection in a national surveillance network, PulseNet USA

**DOI:** 10.3389/fmicb.2023.1254777

**Published:** 2023-09-21

**Authors:** Molly M. Leeper, Beth M. Tolar, Taylor Griswold, Eshaw Vidyaprakash, Kelley B. Hise, Grant M. Williams, Sung B. Im, Jessica C. Chen, Hannes Pouseele, Heather A. Carleton

**Affiliations:** ^1^Division of Foodborne, Waterborne, and Environmental Diseases, Centers for Disease Control and Prevention, Atlanta, GA, United States; ^2^BioMérieux, Sint-Martens-Latem, Belgium

**Keywords:** *Salmonella*, cgMLST, wgMLST, hqSNP, surveillance, epidemiology, silhouette method

## Abstract

*Salmonella enterica* is a leading cause of bacterial foodborne and zoonotic illnesses in the United States. For this study, we applied four different whole genome sequencing (WGS)-based subtyping methods: high quality single-nucleotide polymorphism (hqSNP) analysis, whole genome multilocus sequence typing using either all loci [wgMLST (all loci)] and only chromosome-associated loci [wgMLST (chrom)], and core genome multilocus sequence typing (cgMLST) to a dataset of isolate sequences from 9 well-characterized *Salmonella* outbreaks. For each outbreak, we evaluated the genomic and epidemiologic concordance between hqSNP and allele-based methods. We first compared pairwise genomic differences using all four methods. We observed discrepancies in allele difference ranges when using wgMLST (all loci), likely caused by inflated genetic variation due to loci found on plasmids and/or other mobile genetic elements in the accessory genome. Therefore, we excluded wgMLST (all loci) results from any further comparisons in the study. Then, we created linear regression models and phylogenetic tanglegrams using the remaining three methods. K-means analysis using the silhouette method was applied to compare the ability of the three methods to partition outbreak and sporadic isolate sequences. Our results showed that pairwise hqSNP differences had high concordance with cgMLST and wgMLST (chrom) allele differences. The slopes of the regressions for hqSNP vs. allele pairwise differences were 0.58 (cgMLST) and 0.74 [wgMLST (chrom)], and the slope of the regression was 0.77 for cgMLST vs. wgMLST (chrom) pairwise differences. Tanglegrams showed high clustering concordance between methods using two statistical measures, the Baker’s gamma index (BGI) and cophenetic correlation coefficient (CCC), where 9/9 (100%) of outbreaks yielded BGI values ≥ 0.60 and CCCs were ≥ 0.97 across all nine outbreaks and all three methods. K-means analysis showed separation of outbreak and sporadic isolate groups with average silhouette widths ≥ 0.87 for outbreak groups and ≥ 0.16 for sporadic groups. This study demonstrates that *Salmonella* isolates clustered in concordance with epidemiologic data using three WGS-based subtyping methods and supports using cgMLST as the primary method for national surveillance of *Salmonella* outbreak clusters.

## Introduction

Non-typhoidal *Salmonella* causes an estimated 1.28 million illnesses, 19,300 hospitalizations, and 380 deaths in the United States every year (Scallan et al., 2015). *Salmonella* infections often cause mild to severe gastroenteritis with diarrhea, fever, and stomach cramps, and invasive infections can be potentially life threatening (Centers for Disease Control and Prevention, 2011). While most people with *Salmonella* infections recover completely, some people may experience long term complications, such as reactive arthritis or inflammatory bowel syndrome after their infection ends (Ternhag et al., 2008; Zha et al., 2019). Contaminated food, water, and contact with infected animals are the sources for most *Salmonella* infections (Scallan et al., 2011) and the number of cases attributed to zoonotic (animal to human) transmission of *Salmonella* has increased in recent years (Dróżdż et al., 2021).

*Salmonella* is a diverse group of bacteria consisting of two species, *Salmonella bongori* and *Salmonella enterica*. *Salmonella enterica* is further classified into six subspecies: *enterica* (I), salamae (II), arizonae (IIIa), diarizonae (IIIb), houtenae (IV), and indica (VI) (Porwollik et al., 2004). *S. enterica* subspecies *enterica* (I) strains represent the majority of *Salmonella* strains isolated from humans and warm-blooded animals, while the other subspecies and *S. bongori* are more typically (though not exclusively) isolated from cold-blooded animals. Of the nearly 2,600 known *Salmonella* serotypes, approximately 50 account for 99% of all clinical isolates of *Salmonella* from humans and domestic mammals in the United States. These 50 serotypes all belong to *S. enterica* subspecies I (Centers for Disease Control and Prevention, 2011; Desai et al., 2013).

PulseNet USA is the national molecular subtyping network for foodborne disease surveillance in the United States (Swaminathan et al., 2001; Gerner-Smidt et al., 2006; Ribot and Hise, 2016). This network detects local and national clusters of foodborne illness, including illnesses caused by *Salmonella*. The rapid detection of illness clusters reduces the likelihood of outbreaks becoming large and widespread, thus preventing illnesses and reducing healthcare costs (Scharff et al., 2016). PulseNet USA consists of over 80 state and local public health laboratories and food regulatory federal agencies coordinated by the US Centers for Disease Control and Prevention (CDC) and the Association of Public Health Laboratories (APHL) (Tolar et al., 2019). National outbreak cluster detection and laboratory support of foodborne outbreak investigations are the principal functions of the PulseNet USA network.

Between 1996 and 2019, pulsed-field gel electrophoresis (PFGE) was the primary subtyping method used by PulseNet participating laboratories. However, due to the limitations of PFGE, including its inability to fully distinguish outbreak cases from background infections for clonal organisms such as some *Salmonella* serotypes (Deng et al., 2014), PulseNet began transitioning to using whole genome sequencing (WGS) as the primary tool for outbreak cluster detection for *Salmonella*, and completed the transition for *Salmonella* in July 2019 (Brown et al., 2019; Ribot et al., 2019; Tolar et al., 2019). WGS provides greater resolution compared with PFGE, resulting in more solved outbreaks with fewer cases and allows multiple characterizations of isolates using a single method (Jackson et al., 2016; Besser et al., 2018; Tolar et al., 2019). For example, WGS analysis can characterize bacteria by identifying the species, serotype, genotype, and resistance genes all within a single laboratory workflow (Stevens et al., 2022). For species identification, an average nucleotide identify (ANI) (Thompson et al., 2013; Yoon et al., 2017) calculation is integrated within a BioNumerics v7.6.31 (Biomérieux, 2022). Reference Identification database. This ANI calculation performs a computational analysis that defines the species within the BioNumerics software. For serotype determination, an *in silico* data analysis tool, SeqSero^1^ (Zhang et al., 2019), has been integrated into PulseNet organism-specific BioNumerics databases, and ResFinder^2^ (Florensa et al., 2022) and PointFinder^3^ (Zankari et al., 2017) have been integrated for resistance profiling. PulseNet-participating laboratories perform WGS on isolates of *Salmonella* and upload analyzed WGS data, including assemblies and metadata, in real time to the PulseNet *Salmonella* national BioNumerics database housed at the CDC, where national outbreak detection takes place (Tolar et al., 2019).

As next generation sequence technology has advanced, surveillance networks such as PulseNet have utilized single-nucleotide polymorphisms (SNPs) and core and whole genome multilocus sequence (cg/wgMLST) typing methods to facilitate phylogenetic analysis between bacterial strains. SNP and MLST-based approaches are used to identify sequences that are genetically related and may have a common source within the context of a foodborne outbreak (Katz et al., 2017; Timme et al., 2017; Stevens et al., 2022). For SNP comparisons, single-nucleotide changes are used to infer phylogenetic relatedness between strains relative to a closely related reference sequence (Katz et al., 2017). Core genome (cgMLST) comparisons examine differences in core genome loci of the isolates (those loci found in at least 95–98% of the reference organism strains used to build the allele scheme) and can be used to generate a phylogeny based on a subset of genes. For whole genome MLST (wgMLST) comparisons, differences in both the core and accessory genome loci of the isolates are compared between strains and their pairwise genomic distances are used to generate a phylogeny (Cody et al., 2017; Pearce et al., 2018; Joseph et al., 2020). Both whole and core genome allele-based approaches are well suited for quickly clustering clinical isolates that may be part of the same outbreak before using higher resolution approaches such as SNP analysis (Stevens et al., 2022). Table 1 provides a comparison of the WGS-based analysis methods used in this study.

**TABLE 1:** Comparison of MLST and SNP-based approaches used in study to assess genetic similarity between genomes.

Comparison of WGS-based typing methods used in study			
**Method**	**Approach**	**Reference**	**Genomic comparison result**
cgMLST	Alignment to scheme of core loci found to be present in ≥ 98% of the representative *Salmonella* genomes used to build the EnteroBase (Achtman et al., 2012) allele scheme (*n* = 3,002)	Repository of allele calls for a set of core loci	Allele distance matrix; UPGMA phylogenetic tree
wgMLST (chromosome-associated loci)	Alignment to a scheme of all chromosome-associated loci (including core) (*n* = 22,457)	Repository of allele calls for a set of chromosome-associated loci	Allele distance matrix; UPGMA phylogenetic tree
wgMLST (all loci)	Alignment to scheme of all 22,457 chromosomal and an additional 2,901 plasmid loci, as well as 7-gene MLST loci (*n* = 25,365)	Repository of allele calls for a set of chromosome-associated loci and accessory loci, and 7-gene MLST scheme (Achtman et al., 2012)	Allele distance matrix; UPGMA phylogenetic tree
hqSNP	Mapping to reference genome; phage regions and plasmid contigs were masked	Closely related reference genome; [SNPs with less reliability were filtered out to produce a list of high quality SNPs (hqSNPs)]	SNP alignment and SNP distance matrix; maximum-likelihood phylogenetic tree

Adapted from Uelze et al. (2020).

For SNP comparisons, CDC has developed a high quality single-nucleotide polymorphism (hqSNP) pipeline called Lyve-SET^4^ (Katz et al., 2017) to compare bacterial strains from foodborne pathogens. This method has been used in numerous foodborne outbreak investigations to examine the phylogeny of strains within an outbreak. The design of Lyve-SET was optimized for epidemiologic investigations and has shown that as phylogenetic relatedness between strains increases, the likelihood of epidemiological concordance increases (Katz et al., 2017).

**FIGURE 1:**
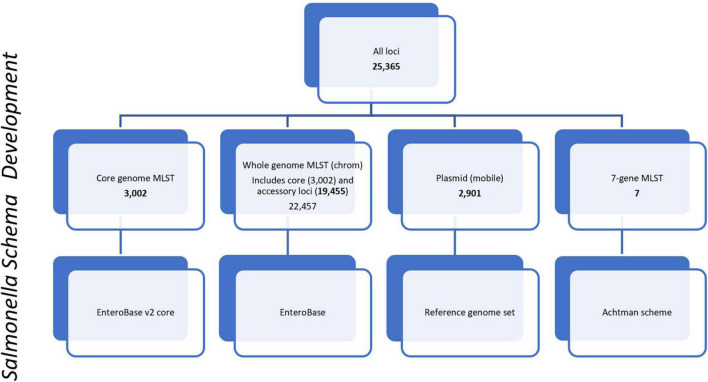
PulseNet *Salmonella* schema development. Number of loci included within schemes are shown for core genome, core plus accessory genome [wgMLST(chrom)], plasmid (mobile), and 7-gene MLST. Loci names are provided in Supplementary Table 1.

For allele-based phylogenomic comparisons, PulseNet primarily uses two allele-based MLST schemes that are incorporated into the *Salmonella* national BioNumerics database. These include the core scheme that contains 3,002 loci and represents the genes most commonly found in subspecies I of *S. enterica*. The core and accessory genome make up the whole genome MLST (chrom) scheme that contains 22,457 chromosomal loci, inclusive of the 3,002 core genome loci (Figure 1). The core and a portion of the whole genome MLST (chrom) schemes used by PulseNet USA were developed by EnteroBase^5^ (Achtman et al., 2012, 2021; Alikhan et al., 2018). Containing over 300,000 *Salmonella* genomes, EnteroBase provides a global overview of the population structure of *Salmonella* and its species and subspecies (Alikhan et al., 2018; Achtman et al., 2021). The remainder of the whole genome MLST scheme was developed by Applied Maths (Biomérieux, 2022) and schemes were integrated into a BioNumerics v7.6.3 database. The wgMLST (all loci) scheme also contains an additional 2,901 plasmid loci {[plasmid loci were not included in the wgMLST (chrom) scheme]}, as well as a 7-gene MLST loci scheme (Achtman et al., 2012; Figure 1). Loci were defined as plasmid loci if the locus occurred 3 or more times in a plasmid, where plasmids were defined as such by RefSeq, the National Center for Biotechnology Information (NCBI) Reference Sequence Database^6^ (Pruitt et al., 2007). Plasmid loci were subsequently removed from the whole genome MLST (chrom) loci scheme. Loci names for all schemes are included in Supplementary Table 1.

Currently, allele-based comparisons are the standard method for detecting outbreak clusters of *Salmonella* illness within PulseNet USA. To accelerate national outbreak cluster detection, sequenced isolates (including both assemblies and metadata) are uploaded to the *Salmonella* national BioNumerics database where they are quickly compared based on their cgMLST allele calls (Tolar et al., 2019; Stevens et al., 2022). While wgMLST is used to further refine clusters of other PulseNet organisms, such as *Listeria* and *Campylobacter* (Nadon et al., 2017; Besser et al., 2018), for *Salmonella*, PulseNet’s primary national cluster definition is seven or more clinical cases (three or more clinical cases for rarer serotypes) uploaded to the *Salmonella* national BioNumerics database within 60 days of each other and each clinical case related within 0–10 allele differences by cgMLST. “Rarer” serotypes comprise those serotypes that fall outside of the top ten serotypes based on frequency of upload to the PulseNet national *Salmonella* database. The number of clinical cases (7 vs. 3), 60-day window, and 0–10 allele difference range, are all elements of PulseNet’s pre-defined cluster coding criteria that have been established based on previous outbreaks. Evaluation of allele differences and phylogenies among genomic profiles that are housed within a central database enables *Salmonella* outbreak cluster detection to take place at the national level.

In addition to the various WGS-based analysis methods used to detect closely genetically related outbreak clusters, previous studies have shown that unsupervised machine learning techniques can be used to cluster genomic data and to create models for source attribution analyses (Coipan et al., 2020; Munck et al., 2020). For genomic clustering, one such technique is K-means analysis, which divides objects into clusters that share similarities and that are dissimilar to objects belonging to another cluster (Rahbar, 2017). This clustering of data can be performed without a predefined cluster threshold such as PulseNet’s national cluster detection definition, and thus, independently of knowledge from prior outbreaks (Coipan et al., 2020).

The objective of this study was to evaluate the concordance of four different WGS-based subtyping methods, including hqSNP, wgMLST (examining all loci or chromosome-associated loci only), and cgMLST, using pairwise genomic differences, linear regression models, phylogenetic comparisons, and K-means analysis as an unsupervised machine learning approach. Our findings may be used to establish allele-based subtyping methods as a validated mechanism for *Salmonella* outbreak cluster detection within a national network such as PulseNet. Additionally, our study aims to demonstrate that the allele schemes built into the PulseNet *Salmonella* national BioNumerics database can be used to reliably detect *Salmonella* outbreak clusters at various levels of genetic diversity with the same epidemiologic concordance as hqSNP, which is currently considered to be a gold standard genomic comparison technique.

## Materials and methods

### Isolate characterization by WGS

Isolate sequences used in this study were generated by PulseNet-participating laboratorians using Illumina MiSeq sequencers (San Diego, CA, USA) according to PulseNet WGS protocols^7^ and with Illumina Nextera XT or DNA Prep library preparation kits (San Diego, CA, USA). Sequence reads were assembled using SPAdes v3.7^8^ (Prjibelski et al., 2020) implemented in BioNumerics v7.6.3 and ANI was used to determine the genus and species of the isolate (genome coverage > 70% and ANI score > 92%) (Thompson et al., 2013). Sequences were checked for contamination using microwell displacement amplification system (MIDAS) (Nayfach et al., 2016), and if another genus was detected at ≥ 1x coverage the sequence was determined to be contaminated and no further analysis was performed. If no contamination was detected, reads were evaluated for quality (average coverage ≥ 30x; average quality score ≥ 30; assembly length = 4.4–5.7 Mbp) in BioNumerics v7.6.3.

**FIGURE 2:**
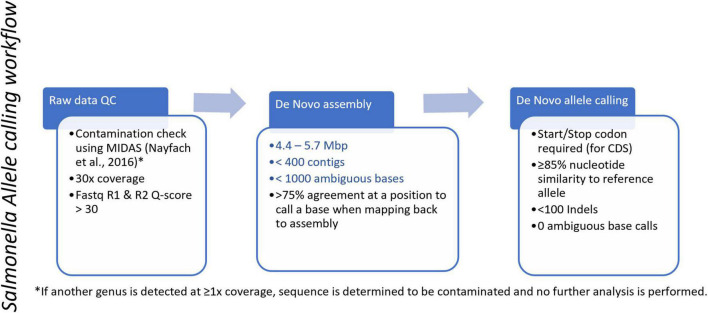
PulseNet *Salmonella* allele calling workflow. Blue text indicates conditional cutoffs.

Once genomes were determined to be *Salmonella* and passed the quality assessment, allele calling was performed. Potential alleles from query genomes were compared to an allele database containing cgMLST, wgMLST, and 7-gene MLST loci using both assembly-free and assembly-based approaches. If the query allele was an exact match to an existing allele in the database, that allele number was assigned to the query allele. If the query allele was not an exact match, then a novel allele could be called by the assembly-based workflow if the query allele contained start and stop codons, no more than 100 base pairs (bp) of insertions and deletions (indels), [as allele calling performance in BioNumerics may be negatively influenced by coding sequence disruptions, including repetitive indels (Weigand et al., 2021)], and ≥ 85% nucleotide similarity to the reference allele for that locus (Figure 2). Reference allele sequences used to call new alleles per locus are included in the *Salmonella* wgMLST Reference Alleles supplementary data file appended to this study. Further quality assessment of genomes was performed using percentage of alleles called in cgMLST, and genomes below 85% of alleles called in the cgMLST scheme were considered to fail quality. While cgMLST schemes generally include those loci present in the majority (95–98%) of isolates in a given group of bacteria (Maiden et al., 2013; Moura et al., 2016; Pearce et al., 2018) in a large national surveillance network such as PulseNet USA, a lower threshold of 85% has been set to adjust for laboratory variability that may be due to differences in staff, capacity, and/or equipment and materials across the network. Sequence types (STs) were determined using a link to EnteroBase’s web-based 7-gene MLST database (Alikhan et al., 2018). Sequences were uploaded to the PulseNet *Salmonella* national BioNumerics database and sequence read archive (SRA) at NCBI under the PulseNet *Salmonella* BioProject, PRJNA230403 (Tolar et al., 2019).

### Selection of isolate datasets

A total of 200 *Salmonella* isolates from 9 foodborne outbreaks was selected from the PulseNet *Salmonella* national BioNumerics database. The 9 outbreaks were identified in 2018 and all had well-characterized sources based on epidemiologic investigations conducted by state public health departments and the outbreak response and prevention branch at the CDC. Each outbreak was assigned a number between 01 and 09, and that number was used to reference the outbreak throughout the study (Table 2). WGS data was available for all 200 outbreak isolates. Since the goal of outbreak detection is to distinguish outbreak-related isolates from non-outbreak isolates circulating at the same time, a selection of sporadic isolates was chosen to assess the ability of the MLST allele schemes to differentiate outbreak isolates from contemporary sporadic isolates. Sporadic isolates were chosen based on matching serotype and/or 7-gene MLST sequence type (ST) when compared to the corresponding outbreak isolates. The sporadic isolates were recovered within the same year as the outbreak isolates, except for one outbreak (outbreak 01) where sporadic isolates from other years were used because there were none available from the outbreak year. Isolates were determined to be sporadic if they were not associated with any previously detected or investigated disease clusters. A total of 47 sporadic isolates were chosen, and the number of sporadic isolates per outbreak varied depending on data availability; i.e., the number of sporadic isolates was lower among more rare serotypes and/or STs. For more common serotypes and/or STs, not all sporadic isolates that matched by serotype and/or ST were included; rather, the selection was limited to contemporary isolates (within a six-month window of the outbreak, based on median collection date) with no previous disease cluster association. WGS data was available for all 47 sporadic isolates.

**TABLE 2:** Summary of information for outbreaks included in study.

Outbreak number (assigned in study)	PulseNet outbreak code*	Total number of outbreak isolates	Confirmed source	Serotype	MLST_ST	Range of collection dates	Total number of sporadic isolates	Sporadic isolate dataset defined by
01	1801MLJIX-1	15	Sprouts	Cubana	ST286	2/5/2018 to 4/25/2018	2	ST, serotype
02	1802MLJBP-1	48	Eggs	Braenderup	ST22	2/28/2017 to 5/07/2018	10	ST, serotype
03	1806NYJPX-1	31	Kosher chicken	I 4, [5], 12:i:-	ST19	9/26/2017 to 8/16/2018	10	ST, serotype
04	1807ORJDX-1	18	Ground beef	Dublin	ST10	7/17/2018 to 07/24/2018	1	ST, serotype
05	1808MLJPX-1	8	Whey protein	Typhimurium	ST19	6/27/2018 to 7/31/2018	11	ST, serotype
06	1809TNJEG-1	54	Eggs	Enteritidis	ST11	3/03/2018 to 9/6/2018	3	ST, serotype
07	1810MLJFX-1	7	English cucumbers	Infantis	ST32	8/27/2018 to 9/17/2018	3	ST, serotype
08	1810MLJRF-1	7	Cake mix	Agbeni	ST2606	6/13/2018 to 10/8/2018	4	ST, serotype
09	1811MLCO2-1	12	Tahini	Concord	ST599	6/19/2018 to 1/6/2019	3	serotype

*PulseNet outbreak codes are designated by the 2-digit year in which the outbreak was detected, 2-digit month in which the outbreak was detected, lab ID/state in which the outbreak was detected (“ML” = multi-state), 3-digit serotype code (Gerner-Smidt et al., 2006), followed by–#. If multiple outbreaks meet the same criteria, then # is changed from 1 to 2, 2 to 3, etc. For example, 1712MLJPX-2 represents the 2nd multi-state Typhimurium outbreak detected in December 2017. 1712MLJPX-3 represents the 3rd multi-state Typhimurium outbreak detected in December 2017, and so on.

### High quality SNP analysis

High quality SNP (hqSNP) data was generated for all outbreak and sporadic isolates included in the study. The hqSNP analyses were generated through the SNP-calling pipeline Lyve-SET v.1.1.4f with the default modules selected for mapping and SNP calling (see text footnote 4). Prior to SNP calling, options were set according to the *Salmonella*-specific thresholds specified under the “*Salmonella_enterica*” configuration; Lyve-SET workflow option “–presets,” respectively (Katz et al., 2017). An internal draft reference, belonging to the specified outbreak, or an external closed reference, neither associated with the outbreak or sporadic isolate set, was selected (Supplementary Table 2). Reference sequences were assembled using SPAdes v.3.14.0 with plasmids masked using PlasFlow v1.1^9^ (Krawczyk et al., 2018) through identification and exclusion. Phages were masked using the Lyve-SET workflow. For each outbreak, two hqSNP analyses were performed where one contained solely outbreak associated genomes and the second included the sporadic set for the outbreak. A phylogenetic tree (RaxML) (Stamatakis, 2014) and pairwise SNP difference matrix were generated for each hqSNP analysis.

### Comparison of WGS-based subtyping methods

For this study, pairwise genomic differences, linear regression models, phylogenetic tanglegrams, and K-means analysis were used to evaluate the concordance between SNP and allele-based subtyping methods. To generate pairwise genomic differences for the allele-based methods, a cluster analysis in BioNumerics v7.6.3 was performed for each outbreak using wgMLST [all loci (chromosomal plus plasmid)], wgMLST [chrom (chromosome-associated loci only)], and cgMLST (core loci), with unweighted pair group method and arithmetic mean (UPGMA) used as the clustering technique. For each outbreak, allele differences were exported into pairwise matrices. For two outbreaks where there were large discrepancies in pairwise differences observed when wgMLST (all loci) was used, the number of isolates aligned to plasmid-associated loci was identified using the sub-scheme of 2,901 plasmid-associated loci (Figure 1) available in BioNumerics v7.6.3. Due to these discrepancies, the wgMLST (all loci) scheme was excluded from further analysis for the remainder of the study. Pairwise genomic differences for SNP analyses were evaluated using the SNP difference matrices generated for each outbreak. The overall range of SNP or allele differences between strains was recorded for each outbreak.

For the regression models, pairwise genomic difference matrices for the outbreaks were combined into one overall profile per subtyping method for cgMLST, wgMLST (chrom), and hqSNP. Pairwise differences were calculated within outbreaks, rather than between outbreaks, before being combined into the overall profiles. Using cgMLST, wgMLST (chrom), and hqSNP, three scatterplots were created in R/R Studio v1.4.1717 using the *ggplot2* package to compare the genomic differences generated by one subtyping method to that of the other two. For each scatterplot, a simple linear regression line was fitted to model the relationship between methods.

For the tanglegrams, outbreak isolates were combined with their corresponding sporadic isolate sets and constructed into dendrograms of the clustering phylogeny. Three tanglegrams were generated for each outbreak, whereby dendrograms obtained using each subtyping method, [cgMLST, wgMLST (chrom), and hqSNP] were compared to that of the other two. Allele-based dendrograms were constructed in BioNumerics v7.6.3 using UPGMA as the clustering technique. SNP-based dendrograms were constructed using the maximum likelihood method. All allele and SNP-based dendrograms were converted to Newick format and assembled into tanglegrams in Base R v4.1.2 (R Core Team, 2023) using the *dendextend* package and the layout was optimized using the *step2side* method (Galili, 2015).

For the K-means analysis, pairwise genomic differences from outbreak isolates were combined with that of corresponding sporadic isolates for 8 of the 9 outbreaks. Outbreak 04 was excluded from the K-means analysis because it contained only one isolate in the sporadic isolate set, preventing a separate group of sporadic isolates from forming. For each combined set of outbreak and sporadic isolate pairwise genomic differences, the silhouette method (Shutaywi and Kachouie, 2021) in R/R Studio v1.4.1717 (*Nbclust* package) (Charrad et al., 2014) was used to identify the optimal number of clusters, or K, in each set. Then, a hierarchical divisive cluster analysis was performed in R/R Studio v1.4.1717 using the *hclust* function (*Stats* package) (R Studio Team, 2023) to show partitioning of outbreak and sporadic isolates into two groups per outbreak. The *fviz_dend* and *fviz_cluster* functions (*Factoextra* package) (Kassambara and Mundt, 2020) were used for data visualization. This exercise was performed for each combined outbreak and sporadic isolate set using cgMLST, wgMLST (chrom), and hqSNP pairwise genomic differences.

### Statistical evaluation of concordance between subtyping methods

For the regression models, the *lm()* function in R/R Studio v1.4.1717 (*R stats* package) (R Core Team, 2023; R Studio Team, 2023) was used to return information about the strength of each model as measured by an *R*^2^ value. Additionally, a Pearson’s correlation coefficient was calculated (*Performance Analytics* package) to show the overall correlation between each subtyping method. For the tanglegrams, the association of the branches in the two opposing dendrograms was statistically measured by calculating the Baker’s gamma index (BGI) (Baker, 1974) and cophenetic correlation coefficient (CCC) (Saraçli et al., 2013). Both measures were obtained using the *dendextend* package in Base R v4.1.2 (R Core Team, 2023). For the K-means analysis, the *Km_stats* function from the *cluster* and *NbClust* packages in R/R Studio v1.4.1717 were used to obtain a Pearson’s gamma coefficient to describe the statistical significance of *K* = 2, once 2 had been defined as the optimal number of clusters in each combined set of outbreak and sporadic isolates based on the maximum silhouette score.

## Results

### Summary of outbreak information

A summary table of the outbreaks included in this study is shown in Table 2. Outbreaks included 9 unique serotypes. Collection dates ranged from 2/28/2017 to 10/08/2018. For 8 of 9 outbreaks, sporadic isolate sets were defined using both serotype and ST; for one outbreak, only serotype was used.

### Pairwise differences

For each outbreak, the range of SNP and allele-based pairwise genomic differences between strains is shown in Table 3. SNP differences were highly concordant with cgMLST and wgMLST (chrom) allele differences. However, SNP, cgMLST, and wgMLST (chrom) allele differences were less concordant when compared to wgMLST (all loci) differences. For 2 outbreaks, (outbreaks 03 and 04), there were much larger allele difference ranges using wgMLST (all loci) compared to SNP and cgMLST allele difference ranges. For outbreak 03, cgMLST allele differences ranged from 0–4 and wgMLST (all loci) differences ranged from 0 to 66; SNP differences ranged from 0 to 8. For outbreak 04, cgMLST allele differences ranged from 0 to 0 and wgMLST (all loci) differences ranged from 0 to 62; SNP differences ranged from 0 to 0. Within outbreak 03, there were 7 isolates that extended wgMLST (all loci) differences out to the higher range, of which 4 contained allele differences that were aligned to plasmid-associated loci. Within outbreak 04, there was 1 isolate that extended the wgMLST (all loci) difference range, and this isolate contained allele differences aligned to plasmid-associated loci. For these 2 discrepant outbreaks, pairwise SNP differences were highly concordant between cgMLST and wgMLST (chrom) but not with wgMLST (all loci). Due to these discrepancies, the wgMLST (all loci) scheme was not analyzed further.

**TABLE 3:** Range of hqSNP, cgMLST, and wgMLST allele differences between strains for the outbreaks included in this study.

	Range of pairwise distances by typing method					
**Outbreak number assigned in study**	**SNP**	**cgMLST**	**wgMLST (chromosomal loci)**	**wgMLST (all loci)**	**Total number of isolates extending allele difference range by wgMLST (all loci)**	Number of outbreak isolates with allele differences aligned to plasmid-associated loci* (isolate identifiers)
01	0–14	0–9	0–10	0–12		
02	0–33	0–21	0–28	0–28		
03	0–10	0–4	0–8	0–66	7	4 (PNUSAS054137, PNUSAS051669, PNUSAS038526, PNUSAS037576)
04	0–0	0–0	0–0	0–62	1	1 (PNUSAS049574)
05	0–7	0–3	0–5	0–5		
06	0–17	0–10	0–14	0–14		
07	0–0	0–0	0–1	0–3		
08	0–4	0–5	0–6	0–6		
09	0–7	0–4	0–7	0–7		

For wgMLST allele differences, ranges are shown for both the chromosomal loci and all loci. Two outbreaks with extended allele difference ranges by wgMLST (all loci) are highlighted in gray, and, within these outbreaks, the number of isolates aligned to plasmid-associated loci is shown. The background color shading indicates the two outbreaks with isolates aligned to plasmid-associated loci.

### Linear regression models

**FIGURE 3:**
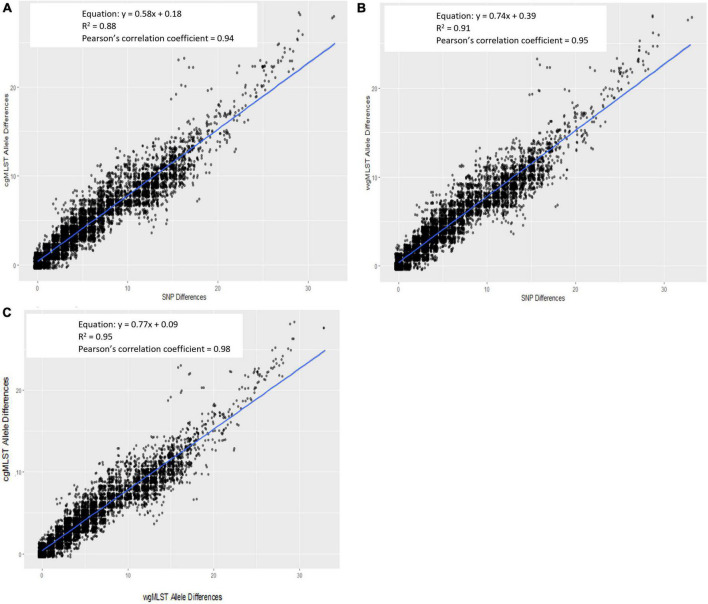
Scatterplot of Lyve-SET hqSNP differences against cgMLST **(A)** and wgMLST (chrom) **(B)** pairwise allele differences and wgMLST (chrom) against cgMLST pairwise allele differences **(C)**. Regression equations, *R*^2^ values, and Pearson’s correlation coefficients are displayed on plots.

For all outbreak isolate sequences, allele-based [cgMLST and wgMLST (chrom)] pairwise genetic differences were plotted against their respective hqSNP differences and are shown in Figure 3A (cgMLST) and Figure 3B [wgMLST (chrom)]. The slopes of their linear regressions were 0.58 and 0.74, respectively, indicating that there were slightly lower allele differences between pairwise isolates versus hqSNP differences. The y-intercepts comparing cgMLST and wgMLST (chrom) allele differences to hqSNP differences were 0.18 and 0.39, respectively, illustrating that on average, sequences that were zero hqSNPs different were < 1 allele different. The goodness of fit for these models, as measured by an *R*^2^ value, were 0.88 (cgMLST vs. hqSNP) and 0.91 [wgMLST (chrom) vs. hqSNP]. The Pearson correlation coefficients were 0.94 for cgMLST and 0.95 for wgMLST (chrom) compared to hqSNP analysis. For the same set of outbreak isolate sequences, there was also high correlation (*R*^2^ = 0.95) between cgMLST and wgMLST (chrom) allele differences. The slope of the linear regression was 0.77, indicating that there were slightly lower cgMLST allele differences per wgMLST (chrom) allele differences, as expected. The y-intercept was 0.09, illustrating that sequences that were zero alleles different by wgMLST using the chromosomal loci scheme were < 1 allele different by cgMLST. The Pearson correlation coefficient was 0.98 for cgMLST compared to wgMLST (chrom) allele differences (Figure 3C). In all three regression plots, we observed a shift in the proportion of points falling below the regression line at approximately 12–15 genomic differences on the *x*-axis. This may be a reflection of the size and genetic diversity of outbreaks 02 and 06, both of which contained a larger number of outbreak sequences relative to other outbreaks (Table 2) as well as wider SNP and allele ranges relative to other outbreaks (Table 3).

### Tanglegrams

**FIGURE 4:**
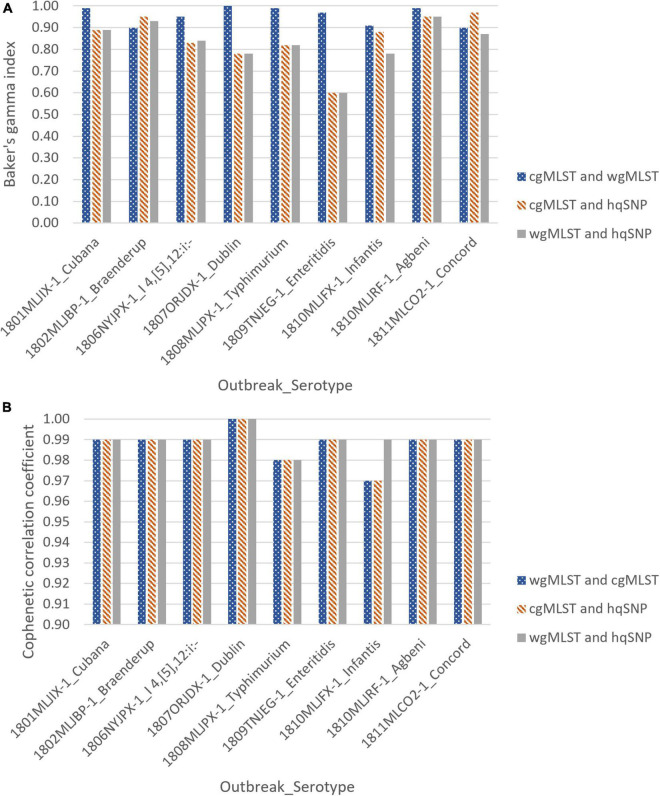
Baker’s gamma indices **(A)** and cophenetic correlation coefficients **(B)** for outbreak tanglegrams.

For all 9 outbreaks, tanglegrams showed high concordance between the subtyping methods in terms of each method’s ability to cluster outbreak and sporadic isolates separately. Clustering concordance of allele-based methods [cgMLST vs. wgMLST (chrom)] when compared to one another was measured with a Baker’s gamma index (BGI) between 0.90 and 1.00, indicating statistically similar clustering between trees. There was moderate to high clustering concordance for allele vs. hqSNP tanglegrams, with BGI values ranging from 0.60 to 0.97 for cgMLST and 0.60 to 0.95 for wgMLST (chrom). Across all subtyping methods and for all 9 outbreaks, the cophenetic correlation coefficient was ≥ 0.97, representing high preservation of original pairwise distances in the dendrograms (Supplementary Table 3 and Figure 4).

### K-means analysis

**FIGURE 5:**
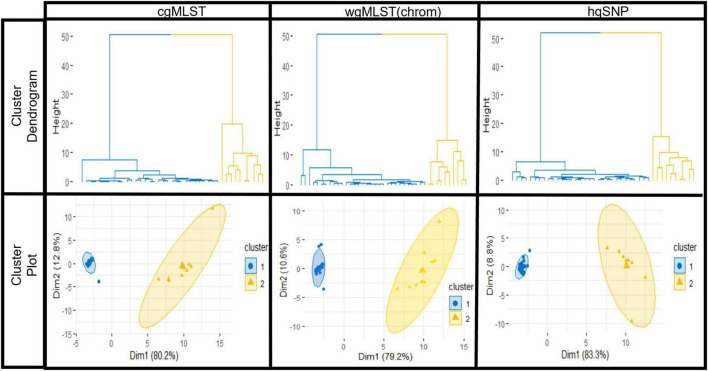
K-means analysis results for one representative outbreak (outbreak 03) using cgMLST, wgMLST (chrom), and hqSNP pairwise genomic differences. Top row: hierarchical clustering results of the dataset show partitioning of outbreak (blue) isolate sequences and sporadic (orange) isolate sequences. Bottom row: elliptical cluster plots show outbreak (blue) and sporadic (orange) isolates plotted separately on a two-dimensional plane, with ellipses fit to the points in the two clusters. On elliptical plots, the sum of values on the *x* and *y*-axis scales indicate that a principle component analysis (Ding and He, 2004) accounts for 93.0% (cgMLST), 89.8% [wgMLST(chrom)], and 92.1% (hqSNP) of variation.

For all outbreaks and across each subtyping method, [cgMLST, wgMLST (chrom), and hqSNP], the silhouette score was maximized at *K* = 2, designating 2 as the optimal number of clusters or groups within each combined set of outbreak and sporadic isolate sequences. The statistical significance of *K* = 2 was measured by Pearson’s gamma coefficient, which ranged from 0.93 to 1.00 across outbreaks, indicating high quality of clustering performed by the K-means analysis (Supplementary Table 4). For each outbreak, the average silhouette width for the outbreak isolate group was consistently high across subtyping methods and ranged from 0.91 to 0.99 (cgMLST), 0.88 to 0.99 [wgMLST (chrom)], and 0.87 to 0.99 (hqSNP), where a value close to 1.00 indicates more cohesive clustering. The average silhouette widths for the sporadic isolate groups were more variable, ranging from 0.21 to 0.96 (cgMLST), 0.16 to 0.93 [wgMLST (chrom)] and 0.31 to 0.96 (hqSNP). Among the sporadic isolate groups, a lower silhouette width was observed for outbreaks where the sporadic isolate set contained a low number of isolates and/or contained more genetic diversity, preventing isolates from clustering as tightly with one another into their own group (Supplementary Table 5). For all 9 outbreaks, a hierarchical divisive cluster analysis showed optimal partitioning of outbreak and sporadic isolates into two distinct groups across subtyping methods, as shown with cluster dendrograms and elliptical plots. These are displayed for one representative outbreak (outbreak 03) in Figure 5.

Table 4 provides a summary of all metrics obtained for each WGS-based analysis method in this study.

**TABLE 4:** Summary table of metrics obtained in study.

	Regression analysis			Phylogenetic clustering analysis		K-means analysis			
	**Slope equation**	***R* ^2^**	**Pearson’s correlation coefficient**	**Range of BGI values across outbreaks**	**Range of CCC values across outbreaks**		**Range of maximum silhouette scores for *K* = 2 across outbreaks (Pearson’s R for *K* = 2)**	**Range of average silhouette widths for outbreak isolate groups**	**Range of average silhouette widths for sporadic isolate groups**
cgMLST vs. hqSNP	*y* = 0.58x + 0.18	0.88	0.94	0.60–0.97	0.97–1.00	cgMLST	0.81 (0.92)–0.99 (1.00)	0.91–0.99	0.21–0.96
wgMLST (chrom)* vs. hqSNP	*y* = 0.74x + 0.39	0.91	0.95	0.60–0.95	0.99–1.00	wgMLST (chrom)*	0.78 (0.93)–0.99 (1.00)	0.88–0.99	0.16–0.93
cgMLST vs. wgMLST (chrom)*	*y* = 0.77x + 0.09	0.95	0.98	0.90–1.00	0.97–1.00	hqSNP	0.79 (0.94)–0.99 (1.00)	0.87–0.99	0.31–0.96

wgMLST (all loci) was excluded from regression analysis, phylogenetic clustering comparisons, and K-means analysis; only wgMLST(chrom) was used.

## Discussion

Previous studies have shown that as phylogenetic relatedness between bacterial strains increases, the likelihood of epidemiological concordance increases (Katz et al., 2017). Therefore, accuracy in identifying genetically related strains is essential to outbreak detection and investigation. In a large national surveillance network such as PulseNet USA, wherein WGS has become the gold standard for cluster detection of bacterial foodborne pathogens, validation of the methods utilized for routine cluster detection is imperative. Thus, the overall objective of this study was to verify that the whole and core genome MLST-based genomic subtyping methods used within the PulseNet network can be used to reliably detect clusters of illness and to determine if one of the allele-based methods achieves better results than the other. An additional purpose of this study was to determine concordance between a gold standard genomic comparison technique, hqSNP, and the allele-based methods used by PulseNet USA.

The findings of this study indicate that the allele schemes (core genome and whole genome using chromosomal loci) built into the PulseNet *Salmonella* national database generate outputs that are highly concordant with hqSNP analysis and well-aligned with epidemiological data, providing confirmation that allele-based methods can be used to detect clusters of *Salmonella*. This outcome was also observed in a previous study where four well-documented foodborne pathogen events showed concordance between epidemiology and routine phylogenomic analyses (reference-based SNP and wgMLST approaches) (Katz et al., 2017). Additionally, a similar comparative analysis of cgMLST and SNP typing within the context of a European *Salmonella* Enteritidis outbreak again showed comparable findings, wherein cgMLST analysis using the EnteroBase scheme was congruent with the original SNP-based analysis, wgMLST analysis, and epidemiological data (Pearce et al., 2018).

For this study, the concordance between WGS-based subtyping methods was statistically measured by using simple linear regression. These regression models confirmed a direct linear relationship between the outputs of each of three subtyping methods: cgMLST, wgMLST (chrom), and hqSNP. The correlation coefficients quantified the strength of the linear relationship between methods, whereas the regression models expressed their relationship in the form of an equation. This finding is comparable to other studies that have investigated the concordance of WGS-based typing methods using statistical approaches, necessitated by the increasing co-existence of wgMLST and SNP analyses in surveillance of foodborne pathogens (Blanc et al., 2020; Bernaquez et al., 2021). For example, a previous comparison conducted by Bernaquez et al. (2021) of WGS-based subtyping methods was applied to a dataset of *Shigella* isolates and, using linear regression, demonstrated that the amount of allelic differences identified by all MLST-based methods for *Shigella flexneri* was consistent in comparison to the number of high quality single-nucleotide variants (hqSNVs). In our study, while each of the three methods [cgMLST, wgMLST (chrom) and hqSNP] showed high concordance using linear regression, hqSNP was found to be slightly more discriminating than the allele-based approaches, in that there were slightly lower allele differences per hqSNP difference observed in the slopes of the trend lines. This finding is foreseeable, as hqSNP does not depend on a pre-defined scheme; thus SNPs in genomic regions not included in the cgMLST scheme may be detected.

In this study, using the wgMLST (all loci) scheme yielded some discrepancies in allele difference ranges, where for two outbreaks, wgMLST (all loci) allele differences were much higher than the number of pairwise cgMLST or wgMLST (chrom) differences. This discrepancy was also reflected in a previous analysis conducted by Weigand et al. (2021) that showed that while cgMLST and wgMLST pairwise allele differences were concordant among a dataset of *Bordetella pertussis* isolates, wgMLST identified more allelic differences among isolates, reflecting the increased resolution provided by the additional loci, as expected. In the present study, for the two discrepant outbreaks, the larger allele difference range was attributed to one or more isolate sequences that extended the wgMLST (all loci) allele differences beyond cgMLST differences, indicating that the additional differences likely occurred outside of the core genome loci. Indeed, we found that for both discrepant outbreaks in our study, the enhanced discrimination of wgMLST using all loci over cgMLST or wgMLST (chrom) was due to some allele differences that were aligned to plasmid loci in one or more isolate. This observation was also seen in Bernaquez et al.’s (2021) study, where mobile genetic element (MGE)-encoded loci caused inflated genetic variation and discrepant phylogenies for some *Shigella sonnei* outbreaks via wgMLST. In our study, this inflated genetic variation was not observed using hqSNP analysis because these differences likely occurred in plasmid regions that were masked. In a study conducted by Alikhan et al. (2018) a dataset of over 1,000 *Salmonella* Agona genomes from EnteroBase showed more similarity between cgMLST and SNP phylograms than when wgMLST was used, and recommends using cgMLST instead of wgMLST for epidemiological purposes due to the volatility of the accessory genome. Both the present study and the previous studies demonstrated that core genome-based subtyping methods were more phylogenetically consistent and epidemiologically concordant when compared to whole genome-based methods that incorporate all loci, due to their exclusion of genetic variation in the accessory genome.

This analysis provided a visual and statistical phylogenomic comparison of various WGS-based subtyping methods in terms of clustering outbreak and non-outbreak/sporadic isolates. hqSNP and allele-based [cgMLST and wgMLST(chrom)] subtyping methods clustered outbreak and sporadic isolates with similar composition when phylogenies were compared side-by-side through tanglegrams. This concordance was supported by two different statistical indices, the Baker’s gamma index and cophenetic correlation coefficient, where for all outbreaks and across all subtyping methods, BGI values were between 0.60 and 1.00, indicative of statistically similar tree topology that can subsequently be interpreted as strong concordance between workflows (Baker, 1974). Additionally, the cophenetic correlation coefficient was ≥ 0.97 for all outbreaks, demonstrating faithful preservation of the original pairwise genomic distances within the dendrograms (Saraçli et al., 2013). These results conform to what has been observed in previous studies, where visual phylogenomic comparisons concluded that outbreak isolates delimited by both SNP and MLST-based approaches were similarly grouped using phylogenetic trees (Pearce et al., 2018; Jagadeesan et al., 2019; Coipan et al., 2020; Bernaquez et al., 2021; Joseph et al., 2023). Minor differences in cophenetic correlation coefficients between allele and hqSNP-based dendrograms may reflect the differences in clustering techniques used, as allele-based dendrograms were created using UPGMA and SNP-based dendrograms were created using the maximum likelihood method.

To enhance this study, an objective approach, K-means analysis, was used to compare the clustering results of the three different WGS-based subtyping methods as a form of external cluster validation. This was an unsupervised machine learning methodology that was applied to a combined dataset of pairwise genomic differences from outbreak and sporadic isolates to measure their goodness of separation. This technique demonstrated that regardless of the subtyping method used (SNP or allele-based), the combined outbreak and sporadic isolate datasets could be successfully partitioned into two distinct groups. Average silhouette widths were assessed for the outbreak and sporadic groups, where silhouette widths can range from –1.0 to 1.0; a higher value indicating that outbreak sequences were well matched within their own outbreak group and poorly matched to sporadic isolate sequences. This finding parallels a previous analysis that showed that by using an unsupervised machine learning methodology, it is possible to detect an optimal number of clusters that separate outbreak from non-outbreak isolates based on the consensus of the silhouette index, or silhouette score (Coipan et al., 2020). In the present study, the differentiation of outbreak and sporadic isolate sequences was seen across multiple outbreaks of various sizes and serotypes, whereas in previous studies this approach has been limited to a single well-characterized outbreak; however, the overall outcome is consistent across studies. This technique provided a way to measure cluster delineation and goodness of clustering across WGS-based subtyping methods in an objective manner.

Allele-based subtyping methods are well suited to surveillance and outbreak detection since allele calls are stable per isolate, allowing for rapid comparisons once allele calling is performed. Often for hqSNP analyses, parts if not all of the SNP calling pipeline need to be repeated to include additional isolates in an analysis, and this can be labor intensive and delay characterizing new isolates as part of an outbreak. Additionally, performing and interpreting hqSNP analyses requires selecting an appropriate reference sequence and certain technical expertise. However, a disadvantage of allele-based methods is that a sequence error in a locus may lead to an error in allele identification, while SNP approaches can use a variety of filtering methods (for example NCBI’s density filtering^10^ in the SNP tree construction). Additionally, we note that with allele-based subtyping methods, there may be a need to update the schema (i.e., add additional loci) as new sequences are added to the database over time.

As observed in this study as well as in previous analyses, cgMLST may be preferred among allele-based methods for cluster delineation of *Salmonella* isolates due to potential expansions in allele ranges that can occur when using a wgMLST scheme that incorporates accessory or plasmid genome loci. Finally, core genome schemes may be optimal because they enable high resolution within a species which can be used as the foundation of a stable nomenclature that ensures interlaboratory reproducibility and comparisons (Maiden et al., 2013; Moura et al., 2016; Zhou et al., 2017; Joseph et al., 2023). The utility of core genome schemes has been previously demonstrated in other studies that have highlighted the emerging influence of WGS in modern-day outbreak investigations (Cody et al., 2017; Blanc et al., 2020; Joseph et al., 2020; Zhou et al., 2020; Abdel-Glil et al., 2021). Adding to these known advantages, the present study confirms that a cgMLST allele scheme built from EnteroBase can be successfully used for routine surveillance of *S. enterica* with the same epidemiologic concordance as SNP typing methods, facilitating rapid and harmonized genomic comparisons across public health networks.

While this study confirms that the cgMLST allele scheme used by PulseNet USA can be successfully used for routine surveillance of *S. enterica*, we note that many public health networks have been successful in using validated SNP-based cluster detection methods for foodborne disease surveillance. For example, NCBI’s Pathogen Detection Project (PDP) (see text footnote 10), a centralized system that integrates sequence data from bacterial pathogens, quickly clusters related pathogen genome sequences submitted by US and international public health agencies to identify potential disease clusters and possible transmission chains. For each cluster, a phylogenetic tree is reconstructed from the SNPs for that cluster by using the maximum compatibility criteria (Cherry, 2017). The goal of NCBI’s PDP is to identify closely related isolates to aid in real-time outbreak investigations. Since NCBI facilitates the integration of clinical, food, and environmental data using SNP-based trees, which are used for regulatory purposes, PulseNet participating-laboratories must commit to submitting WGS data to NCBI in real-time. A future study could compare in more detail the SNP pipeline used by the US Food and Drug Administration (FDA)’s Center for Food Safety and Applied Nutrition (CFSAN) to the allele-based methods described in this study, as public health researchers may benefit from a more detailed comparison of the two heavily used, well-characterized analytical pipelines. Additionally, our study could have been enhanced by comparing each outbreak cluster to its corresponding SNP cluster on NCBI’s PDP, noting similarities and/or differences in cluster size, inclusivity and exclusivity of isolates, and pairwise distances between clinical and non-clinical samples.

This study has some limitations. One limitation is that sporadic isolate datasets for outbreaks caused by more rare serotypes contained fewer isolates, due to data availability. Additionally, for outbreaks caused by more common serotypes (i.e., Enteritidis, Typhimurium), we did not capture all sporadic isolates that met matching criteria; rather, we limited the sporadic isolate datasets to a selection of contemporary isolates that had not been associated with any previously detected and investigated disease clusters. In true application, the number of sporadic isolates should exceed the number of outbreak isolates by a certain factor, depending on serotype commonality; thus, our datasets may not reflect the true ratio of outbreak versus sporadic isolates in the population. A limitation of the clustering phylogeny comparison is that for allele-based methods, UPGMA was used as the clustering technique and for SNP-based dendrograms, the maximum likelihood method was used. This may have resulted in minor differences in cophenetic correlation coefficients across outbreaks but did not change the overall interpretation. Additionally, we acknowledge that the genetic distances resulting from the hierarchical clustering techniques were fitted to reflect the linkage method used. A limitation of the K-means analysis is that only one method was used to determine the optimal number of groups in the combined outbreak and sporadic isolate datasets, namely, the silhouette method. Other methods used for partitioning datasets into groups or clusters, such as the elbow method and gap static method, were not used in this study primarily because the results we obtained using the silhouette method were not ambiguous, and because the silhouette method has the added advantage of identifying outliers (if present) in a cluster^11^ (Shutaywi and Kachouie, 2021). Finally, the K-means exercise could be repeated in a future study using a larger set of sporadic isolate sequences per outbreak.

## Conclusion

Using nine outbreaks with well-characterized sources, this study confirms that the allele schemes built into the PulseNet *Salmonella* national BioNumerics database provide ideal resolution for detecting outbreak clusters caused by a variety of sources and serotypes. hqSNP and MLST-based [cgMLST and wgMLST (chrom)] analysis of WGS data was highly concordant with epidemiologic information linking cases in *Salmonella* outbreaks and differentiating sporadic isolates that were matched by serotype or 7-gene MLST to outbreak isolates. While pairwise differences were highly concordant between hqSNP, cgMLST, and wgMLST (chrom) analyses, the results showed some discrepancies when wgMLST (all loci) were used, as expected. One explanation for these discrepancies is that the wgMLST (all loci) scheme incorporates the accessory genome loci, including plasmids and mobile genetic elements, while the cgMLST scheme is based on a subset of core loci found to be present in ≥ 98% of the representative *Salmonella* genomes used to build the EnteroBase allele scheme (Achtman et al., 2012). The objective K-means methodology further validates the ability of the allele schemes currently used within PulseNet, particularly for differentiating outbreak and sporadic isolate sequences. While this study shows that allele-based methods can be reliably used for cluster detection, we emphasize that any cluster detection method should be used in conjunction with epidemiologic data gathered from a paired investigation. The findings of this study are substantiated by results of similar preceding studies and should provide public health researchers with additional confidence in using allele-based methods for surveillance of *Salmonella enterica* outbreak clusters.

## Data availability statement

The datasets presented in this study can be found in online repositories. The names of the repository/repositories and accession number(s) can be found in this article/Supplementary material.

## Author contributions

ML: Conceptualization, Data curation, Formal analysis, Investigation, Methodology, Visualization, Writing—original draft, Writing—review & editing. BT: Conceptualization, Data curation, Investigation, Methodology, Writing—review & editing. TG: Formal analysis, Methodology, Writing—review & editing. EV: Formal analysis, Methodology, Writing—review & editing. KH: Writing—review & editing. GW: Methodology, Writing—review & editing. SI: Methodology, Writing—review & editing. JC: Writing—review & editing. HP: Methodology, Software, Writing—review & editing. HC: Conceptualization, Investigation, Methodology, Supervision, Writing—review & editing.

## Footnotes

1
http://github.com/denglab/seqsero2

2
https://cge.food.dtu.dk/services/resfinder/

3
https://bitbucket.org/genomicepidemiology/pointfinder_db/src/master/

4
https://github.com/lskatz/lyve-set

5
https://enterobase.warwick.ac.uk

6
https://www.ncbi.nlm.nih.gov/refseq/

7
https://www.aphl.org/programs/food_safety/pages/pulsenet-international-sops

8
https://cab.spbu.ru/software/spades/

9
https://github.com/smaegol/plasflow

10
https://www.ncbi.nlm.nih.gov/pathogens/faq

11
https://uc-r.github.io/kmeans_clustering
